# Long-Term Patient Satisfaction and Surgical Outcomes With Sophono Implants: A Single-Institution Experience

**DOI:** 10.7759/cureus.72047

**Published:** 2024-10-21

**Authors:** Hesham S Almofada, Marion Atkin, Peter Monksfield, Rupan Banga

**Affiliations:** 1 Otolaryngology - Head & Neck Surgery, King Faisal Specialist Hospital & Research Centre, Riyadh, SAU; 2 Medicine, Alfaisal University, Riyadh, SAU; 3 Ear, Nose and Throat (ENT), University Hospitals Birmingham NHS Foundation Trust, Birmingham, GBR; 4 Osseointegrated Adult Implant, University Hospitals Birmingham NHS Foundation Trust, Birmingham, GBR

**Keywords:** adult, audiological benefit, bone conduction implant, complications, patient satisfaction, percutaneous implants, sophono, transcutaneous implants

## Abstract

Objectives

Percutaneous and transcutaneous bone conduction hearing implants are used in various otological conditions. This study aimed to evaluate patient satisfaction with Sophono implants and their long-term surgical outcomes.

Methods

This retrospective study was conducted at Queen Elizabeth Hospital, Birmingham, between October 2022 and May 2023. Data on the demographics, implant details, patient perception of sound, usage time, and surgical complications were collected from the medical records. Categorical data are presented as frequencies, and numerical data are presented as percentages, ranges, and means.

Results

A total of 15 patients (nine men, six women) with 16 implants (one bilateral) were included. Participants’ ages ranged from 14 to 69 years (mean: 38.6 years). All patients used the device infrequently for various reasons, including feedback, retention, and background noise (11, 8, and 9 patients, respectively). Two patients reported pain at the implantation site after one hour of usage despite adjusting the magnet power. One patient (6.25%) experienced major surgical complications requiring reoperation, and most patients required alternative hearing aids.

Discussion

Sophono implants are passive transcutaneous implants that offer cosmetic advantages and lower skin complications than percutaneous implants. Our findings on the surgical safety profile of Sophono implants are consistent with those reported in the literature. However, in contrast to our study results, most previous studies have reported favorable quality of life and sound perception, especially in long-term users.

Conclusion

Patient satisfaction and audiological benefits of Sophono implants are suboptimal, and the effect on patient satisfaction is questionable.

## Introduction

Bone conduction hearing implants are classified as percutaneous (bone-anchored hearing aid (BAHA)) or transcutaneous (Bonebridge, BAHA attract, and Sophono) [1-4]. They are indicated in children or adults with conditions that prevent conventional behind-the-ear hearing aid use, such as mastoid cavities, mixed hearing loss, moderate-to-severe unilateral or bilateral conductive hearing loss, single-sided deafness, and congenital ear malformation [1-4]. Transcutaneous hearing implants are further divided into passive implants, where vibration is created through intact skin (Sophono), and active implants, whose vibrations are sensed by the cochlea through the skull (Bonebridge) [2,3].

Passive transcutaneous implants require magnetic force to attach the external device to the internal magnet and create vibrations transmitted through the skin and soft tissues [1,3,4]. Transcutaneous implants are more aesthetic than percutaneous implants, with a significantly lower incidence of skin infections than percutaneous implants [1,5]. However, minor skin and soft-tissue complications and major complications requiring explantation have been reported.

Most studies have reported acceptable audiological outcomes; however, the mean follow-up period was only 12-48 months [1,2], and most reported cases of Sophono implants were pediatric (<18 years) [1,2,4-12]. Therefore, this study aimed to evaluate long-term patient satisfaction with Sophono implants and their surgical outcomes in adults.

This manuscript was presented at the British Society of Otology meeting on the 25th of March 2024.

## Materials and methods

Study Design and Inclusion Criteria

This was a retrospective case series study conducted at Queen Elizabeth Hospital Birmingham between October 2022 and May 2023. Ethical approval was granted by the Institutional Audit Committee (CARMS-18756). The study focused on patients who received Sophono transcutaneous bone conduction implants. Inclusion criteria were patients with Sophono implants who had undergone surgery at Queen Elizabeth Hospital Birmingham since the establishment of the program. Patients who received other types of hearing implants or had incomplete records were excluded.

Data Collection

Medical records of patients with Sophono implants were retrieved in collaboration with the Audiology Department. Data collected and retrieved are the demographic data, date of implantation, date of explantation or alternative hearing aid use, patient perception of sound, usage time, and surgical complications were collected.

The cutoff frequency for hearing aid use was defined as >4 hours/day for at least four days of the week. Implants were assessed in the clinic using different assessment methods, for example, scores out of 10 for comfort and clarity in quiet and noisy environments. In addition, feedback, retention, background noise, usage time, and patient complaints were assessed using direct questions.

Statistical Analysis

Descriptive statistics were used to summarize demographic and clinical data. Categorical variables (e.g., presence of complications) were presented as frequencies and percentages. Continuous variables (e.g., age, follow-up duration) were expressed as means and ranges. All statistical analyses were performed using Microsoft Excel (Microsoft Corporation, Redmond, WA).

## Results

A total of 15 patients (nine men, six women) with 16 implants were included in the study; one patient had bilateral implants. Participants’ ages ranged from 14 to 69 years, with a mean of 38.6 years. Five implants were placed on the left side and 11 on the right side (Table 1). The etiology for implantation varied; patients required implantation owing to conductive hearing loss or mixed hearing loss (nine patients), single-sided deafness (three patients), post-vestibular schwannoma (two patients), and congenital ear malformation (one patient) (Table 1).

**Table 1:** Demographic and clinical characteristics of patients implanted with Sophono devices M: Male, F: Female, CHL: Conductive hearing loss, R: right, L: Left

Patient	Age at implantation	Date of implantation	Sex	Side implanted	Aetiology
1	29	20/09/2011	M	R	Right microtia and canal atresia
2	64	03/08/2012	F	L	Single-sided deafness (left)
3	69	04/10/2011	M	R	CHL (mastoid cavity)
4	42	29/09/2011	M	L	Post schwannoma surgery
5	14	10/2012	M	R	CHL (mastoid cavity)
6	23	20/09/2011	F	L	CHL (left)
7	34	28/10/2011	F	R	Mixed hearing loss (right)
8	46	05/04/2011	M	R	Post schwannoma surgery
9	44	05/04/2011	M	R	CHL (right)
10	39	07/03/2012	M	R	CHL (right)
11	51	12/12/2011	F	R	CHL (right)
12	28	07/02/2012	M	L	Single-sided deafness (left)
13	59	20/09/2011	M	R	Single-sided deafness (right)
14	18	17/11/2011	F	R	CHL (bilateral)
				L	
15	34	05/04/2011	F	R	Mastoid cavity (unable to wear hearing aid)

**Figure 1:**
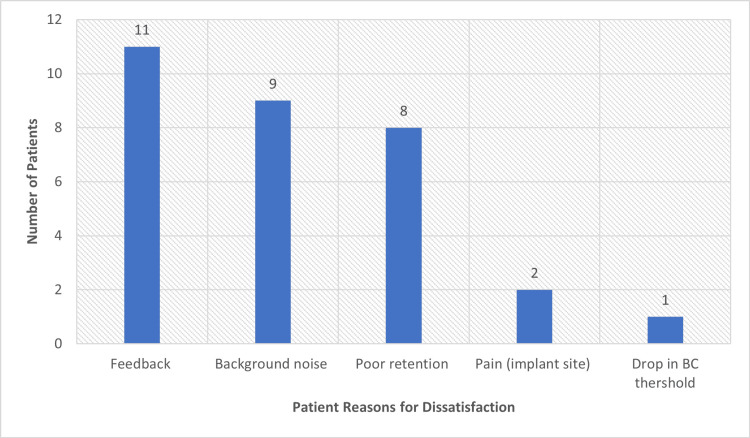
Reasons for dissatisfaction with Sophono implants

Notably, all patients started with the Alpha 1 processor and were upgraded to Alpha 2 during the follow-up period. None of the patients were frequent users for various reasons. The main reasons for discontinuation were feedback (73.3%, 11/15), poor retention (53.3%, 8/15), and issues with background noise (60%, 9/15) (Figure 1). Two patients (13.3%, 2/15) reported pain at the implantation site after one hour of usage despite adjusting the magnet power. One patient (6.6%, 1/15) experienced a significant drop in bone-conduction threshold, which led to dissatisfaction and ineffective aided hearing. One patient continued using the implant, albeit infrequently, despite discontinuation of support by the manufacturer. Most patients obtained limited benefits from the implant, as it affected sound quality and perception and necessitated other procedures or hearing aids (summarized in Table 2). 

**Table 2:** Summary of patient outcomes, complications, and satisfaction factors for Sophono implants

Categories	Number	Percentage
Number of Patients	15	
Number of Implants	16	
Surgical Complication	1/16	6.25%
Outcome (Users vs Non-users)		
Users	NA	NA
Non-users	15/15	100%
Reason for Dissatisfaction (sometimes in combination)		
Feedback	11/15	73.3%
Background Noise	9/15	60%
Poor Retention	8/15	53.3%
Pain at Site	2/15	13.3%
Drop in Bone Hearing Thresholds	1/15	6.6%
Follow-Up Period		
Ranges	1-12 years	
Mean	9.1 years	
Years to Switch to Alternative Hearing Aid		
Range	1-11 years	
Mean	5.8 years	
Alternative Hearing Aid Devices		
BAHA	7/15	46.6%
Cross Hearing Aid	2/15	13.3%
OSIA	1/15	6.6%
Conventional Hearing Aid	4/15	26.6%
Decline	1/15	6.6%

One patient experienced post-operative wound infection and dehiscence that required reoperation and closure. Thus, the major surgical complication rate was 6.25% (1/16). Dissatisfied patients were offered alternative hearing aids of their choice - BAHA (seven patients), Osia (one patient), contralateral-routing hearing aids (two patients), or conventional hearing aids (four patients); one patient declined all the options offered. Patient complaints are summarised in Figure 1. The follow-up period ranged from 1 to 12 years after implantation. The mean follow-up duration was 9.1 years, and the mean number of years to switch to an alternative hearing aid was 5.8 years.

## Discussion

Stenfelt et al. summarised the pathways of sound conduction in bone-conduction hearing implants as follows: (1) sound radiation into the external auditory canal, (2) middle-ear ossicle inertia, (3) cochlear-fluid inertia, (4) cochlear-wall compression, and (5) pressure transmission from the cerebrospinal fluid [13]. Of these pathways, cochlear-fluid inertia seems the most relevant [1-4,13]. Transcutaneous hearing implants can be passive or active [1-4,8]. The Sophono system implant is categorized as a passive transcutaneous implant alongside BAHA attract [1-4,8]. It comprises two connected magnets secured to the skull using five titanium screws and an outer sound processor [3,8]. The external sound processor is attached to the internal device using a magnetic force across the skin. When sound passes through the external device, the vibration is transmitted through the skin, subcutaneous tissue, and two connected magnets to the internal device and five screws in the skull bilaterally [3,8].

Transcutaneous hearing implants are popular because of better cosmesis than percutaneous hearing implants. In addition, skin complications around the abutment, device extrusion, and revision surgery are significantly lesser than those with percutaneous implants [1-3,8].

Sophono implants were the first to be developed in the transcutaneous group, followed by others, such as BAHA attract [8]. Furthermore, Sophono is compatible with 3 Tesla magnetic resonance imaging because it casts a 5-cm shadow, whereas BAHA attract casts an 11-cm shadow [3,8]. Percutaneous implants provide an acoustic gain of approximately 5-7 dB higher than that with transcutaneous implants, pertinent to their direct contact with the skull. In contrast, transcutaneous implants lose some acoustic gain through the skin and subcutaneous tissues with a maximal acoustic gain of approximately 45 dB; the main acoustic loss occurs at high frequencies [3,8].

A systematic review by Cooper et al. identified minor skin and soft-tissue complications in 13.1% of patients with a transcutaneous implant, which resolved spontaneously or by reducing the magnetic force [1]. In contrast, skin complications occur in 9-38% of patients with percutaneous implants [1,3,4]. Furthermore, major complications, defined as any complication that requires active management, such as post-operative seroma, hematoma, wound infections, skin ulcerations, and dehiscence, were reported in 5.2% of patients [1]. The incidence of complications of the percutaneous BAHA system is higher in children than in adults [10], which may have influenced the trend of using transcutaneous implants relatively more frequently in children than in adults.

Another systematic review focusing on Sophono implants by Bezdjian et al. reported a complication rate of 29%, of which 3.5% were major complications that required explantation (two patients with skin breakdown and one patient with severe headache) [2]. The minor complication rate was 24.4% (21/86): moderate-to-severe pain, 9.3% (8/86); pressure necrosis or discomfort, 5.8% (5/86); wound infections requiring medical treatment, 4.7% (4/86); isolated skin erythema, 3.5% (3/86); and skin erythema with ulceration, 2.3% (2/86) [2].

Regarding the surgical complication profile, Sophono implants are safer than percutaneous implants; however, the reported audiological benefits of percutaneous implants are slightly better than those of other transcutaneous implants [1,6,11,14]. Another recent review has reported that passive transcutaneous devices have higher complication rates than active ones (Sophono, 26.6%, and BAHA attract, 24.4%, versus Bonebridge, 11.8%) [5]. The reported complication rates for passive and active transcutaneous devices are approximately 35.7% and 9.4%, respectively [5].

Furthermore, a previous study found no significant difference between Sophono implants and contralateral-routing hearing aids for single-sided deafness [1]. This was reflected in our study, in which most patients were audiologically more satisfied with alternative hearing aids.

In contrast to our findings, most studies have reported favorable quality-of-life and sound-perception outcomes in patients with Sophono implants. However, these studies primarily focus on short- to medium-term follow-up periods, generally less than four years [1,2,4-7,9-12]. Our longer follow-up period (mean: 9.1 years) revealed a trend of decreased device usage and increased dissatisfaction over time, with a mean time to switch to an alternative hearing aid of 5.8 years. This decline in satisfaction may not be fully captured in studies with shorter follow-up periods. A few exceptions, however, have noted a lack of improvement or even deterioration in outcomes [1,6]. However, few exceptional studies failed to identify improvement or deterioration [1,6]. Notably, in contrast to our study, previous studies predominantly included pediatric patients. This could be the reason for the discrepancies between our results and those of previous studies, and future studies on long-term adult outcomes should be considered. Certain limitations of our study should also be noted, namely, the retrospective design and the small and heterogeneous study population.

## Conclusions

Sophono implants are safe and offer a lower soft-tissue complication rate and better cosmesis compared to percutaneous implants; however, our findings suggest that patient satisfaction and audiological benefits can vary considerably. Moving forward, more studies exploring the long-term quality of life and patient satisfaction, as well as those comparing passive and active transcutaneous hearing implants, will be needed.
